# Biliverdin reductase-A attenuated GMH-induced inflammatory response in the spleen by inhibiting toll-like receptor-4 through eNOS/NO pathway

**DOI:** 10.1186/s12974-018-1155-z

**Published:** 2018-04-20

**Authors:** Yiting Zhang, Yan Ding, Tai Lu, Yixin Zhang, Ningbo Xu, Devin W. McBride, Jiping Tang, John H. Zhang

**Affiliations:** 1 Department of Ophthalmology, The First Affiliated Hospital of Chongqing Medical University, Chongqing, China; 2 Department of Physiology and Pharmacology, Loma Linda University School of Medicine, Loma Linda, CA USA; 3 Departments of Neurosurgery and Anesthesiology, Loma Linda University School of Medicine, Loma Linda, CA USA

**Keywords:** Germinal matrix hemorrhage, Biliverdin reductase-A, Toll-like receptor 4, Splenic response, Endothelial nitric oxide synthase

## Abstract

**Background:**

Germinal matrix hemorrhage (GMH) is a common neurologic event with high morbidity and mortality in preterm infants. Spleen has been reported to play a critical role in inflammatory responses by regulating peripheral immune cells which contributes to secondary brain injury.

**Methods:**

The current study investigated the mechanistic role of biliverdin reductase-A (BLVRA) in the splenic response and brain damage in neonates following a collagenase GMH model. Neurological outcomes and splenic weights were assessed. Neutrophil production and infiltration were quantitated in the spleen and brain, respectively. Western blot was performed in both splenic and brain tissues to measure protein levels of toll-like receptor 4 and proinflammatory cytokines.

**Results:**

BLVRA treatment alleviated GMH-induced developmental delay and attenuated splenic atrophy at 1 and 3 days after GMH. Quantification analysis showed that spleen-stored peripheral immune cells mobilized into circulation and infiltrated in the brain following GMH, which was abrogated by BLVRA administration, resulting in reduced splenic inflammatory response. Furthermore, we showed that regulation of eNOS/NO signaling by BLVRA stimulation blunted toll-like receptor-4 (TLR4) signal. The eNOS-generated NO, in part, translocated BLVRA into the nucleus, where BLVRA inhibited TLR4 expression.

**Conclusion:**

We revealed a BLVRA-dependent signaling pathway in modulating the splenic inflammation in response to GMH via the eNOS/NO/TLR4 pathway.

## Background

Germinal matrix hemorrhage (GMH) is a common neurological deficit in premature infants and is strongly related to high mortality and morbidity. It is attributed to the rupture of immature blood vessels in the subependymal (or periventricular) germinal zone in the brain of preterm neonates [1]. In the USA, it affects 12,000 premature newborns each year, which accounts for up to 20% infants before 32 weeks of gestation [2]. Clinical studies have revealed that premature newborns with GMH can develop hydrocephalus or life-long neurologic dysfunction, which include mental retardation, cerebral palsy, and psychiatric disorders [3, 4]. Accumulating evidence shows that stroke can elicit systemic response in multiple organs, rather than being solely a brain injury. Therefore, more studies are needed to assess potential relationship between the brain and peripheral organs.

Spleen is a secondary peripheral immune organ where immune cells were pooled. It plays a role in clearance of dying red blood cells, hemoglobin removal, iron homeostasis, and B cell antibody production. Mounting evidence shows that post-stroke immunological responses are not only generated from brain, but also in the peripheral organs and tissues, including bone marrow, blood, and the spleen. Current studies have investigated splenic responses after stroke and highlighted the effect of the spleen in regulating peripheral immune system which is considerably involved in the secondary brain injury [5]. Furthermore, more and more splenic responses induced by stroke are being investigated, such as sympathetic nervous activation [6], chemotactic cytokines generation [7], and antigen presentation [8]. Once stroke induces splenic contraction, splenic cells mobilize into the circulation and subsequently accumulate in the region of primary brain injury [9]. In addition, splenectomy and irradiation of spleen both successfully attenuate splenic responses induced by brain injury and contribute to decreased brain lesion size and neurodegenerative outcomes [10–12].

Biliverdin reductase-A (BLVRA, also called BVR-A), is a pleiotropic enzyme with the main isoform of biliverdin reductase (BVR). It converts biliverdin-IX-alpha into bilirubin-IX-alpha, which plays a crucial role in the cellular redox cycle [13]. Bilirubin has been showed to be a potent antioxidative neuroprotectant [14–16]. BLVRA has a serine/threonine/tyrosine kinase which modulates insulin signaling and contributes to reducing cognitive dysfunction in Alzheimer’s disease [17]. The modulation of inducible isozyme heme oxygenase-1 (HO-1), which is the upstream of BVR, was involved in multifactorial mechanisms correlated with immunomodulation, circulatory integrity, and cell survival [18]. Upregulation of HO-1 in macrophages led to an anti-inflammatory M2 phenotype with increased interleukin (IL)-10, but decreased tumor necrosis factor (TNF)-α [19, 20]. Furthermore, studies have investigated whether BV administration regulates inflammatory responses in macrophages by inhibiting TLR4, a key regulator of the innate immune system. It has been shown that BV-induced anti-inflammatory effect was BVR-dependent, since secretion of pro-inflammatory cytokines were observed in the absence of BVR in macrophages [21]. Moreover, BV treatment induced anti-inflammatory effect was abolished with endothelia nitric oxide synthase (eNOS) inhibition, even in the presence of TLR4 inhibition. However, whether BLVRA administration attenuates spleen-associated secondary brain injury has not been evaluated.

In this study, we investigated if BLVRA administration inhibited TLR4-mediated splenic immune response in a neonatal rat GMH model. We hypothesized that BLVRA administration induced eNOS phosphorylation and NO generation, leading to the translocation of BLVRA into the nucleus, where BLVRA was a direct inhibitor of TLR4 expression. This would result in attenuated splenic inflammatory responses and alleviated post-hemorrhagic behavioral dysfunction and brain tissue loss.

## Methods

### Animals and surgeries

All studies and procedures were conducted in accordance with the National Institute of Health guidelines for the treatment of animals and were approved by the Loma Linda University Institutional Animal Care and Use Committee. One hundred and thirty-two P7 Sprague-Dawley neonatal rat pups (brain development is comparable with human 30–32 weeks of gestation; 12–15 g; Envigo, Livermore, CA, USA) were used in this study. GMH was induced using bacterial collagenase by a stereotactically guided infusion as previously described [22]. Rat pups were anesthetized with 3% isoflurane while placed onto a stereotaxic frame then isoflurane concentration was reduced to 2%. The scalp area was sterilized by isopropyl alcohol. Bregma was used as a reference point, stereotactic coordinates were measured as follows: 1.6 (rostral) and 1.5 mm (lateral), and 2.8 mm (depth). A 1 mm burr hole was drilled, and then a 27-gauge needle was inserted at a rate of 1 mm/min. 0.3 U clostridial collagenase VII-S (Sigma, St Louis, MO, USA) was infused by the Hamilton syringe. The needle is left in the place for 10 min after injection to prevent “black-leakage.” Once the needle was removed with a rate of 1 mm/min, the bone wax was used to seal the burr hole and then incision site was sutured. After given buprenorphine, animals were allowed to recover on a 37 °C heated blanket. After they were fully recovered, animals were placed back to their dams. Sham surgeries consisted of needle insertion without collagenase infusion.

### Animal treatment

BLVRA recombinant protein was purchased from Abnova. All drugs were injected intranasally to experimental animals at 1 h after GMH at doses of eNOS inhibitor (Iromycin A, 1 mg/kg, Abcam), iNOS inhibitor (Mercaptoethylguanidine dihydrobromide, MEG, 1 mg/kg, Abcam), and NO donor (Sodium nitroprusside, SNP, 0.1 nmol, Sigma-Aldrich). The scrambled siRNA (Origen) and BLVRA siRNA (Origen) were injected intraperitoneally at 24 h before GMH.

P7 rat pups were randomly assigned to the following groups: sham-operated (sham, *n* = 24), GMH + vehicle (vehicle, *n* = 18), GMH + BLVRA (*n* = 18), GMH + scrambled siRNA (*n* = 6), GMH + BLVRA siRNA (*n* = 6), GMH + BLVRA + Iromycin A (*n* = 12), GMH + BLVRA + MEG (*n* = 6), GMH + BLVRA + NPA (*n* = 6), sham + BLVRA (*n* = 6), sham + BLVRA + Iromycin A (*n* = 6), sham + BLVRA + MEG (*n* = 6), sham + BLVRA + NPA (*n* = 6), sham + SNP (*n* = 6), GMH + SNP (*n* = 6).

### Neurobehavioral examination

The effects of GMH and BLVRA treatment on the development of neonates were analyzed using righting reflex and negative geotaxis tests. The behavior tests were evaluated in a blinded fashion daily through day 3.

### Spleen measurements

Spleen sizes were quantified using the length (*L*) and thickness (*T*) in the sagittal plane, and width (*W*) at the splenic hilum in the transverse plane. Three sets of these measurements with the least degree of variations were selected. Final spleen volume was calculated from average using the standard prolate ellipsoid formula. The formula incorporated product of one-dimensional diameters (*W* × *T* × *L*) into the eq. (*W* × *T* × *L* × π/6), where *V* is the ellipsoid volume. We normalized the spleen weight to the whole body weight to get the relative ratio [11]. The spleen/body weight was used to show the variation of spleen weight.

### Western blot

The lysates from tissues were separated by SDS-PAGE then transferred onto nitrocellulose membranes. Membranes were blocked with 5% non-fat milk in Tris-buffered saline containing 0.1% Tween 20. Then membranes were incubated with following primary antibodies: anti-BLVRA (1:1000, Santa Cruz), eNOS (1:1000, Santa Cruz), phospho-eNOS (S1177) (1:1000, Santa Cruz), TLR4 (1:1000, Abcam), Actin (1:3000, Abcam), 3-Nitrotyrosine (3-NT, 1:1000, Abcam), 4-Hydroxynonenal (HNE, 1:1000, Abcam), dinitrophenol (DNP, 1:1000, Abcam), IL-1β (1:1000, Abcam), IL-6 (1:2000, Abcam), TNF-α (1:1000, Abcam), Lamin B (1:1000, Abcam) at 4 °C overnight followed by appropriated secondary antibodies (1: 5000, Santa Cruz) for 2 h. Immunocomplexes were visualized by ECL ECL plus Kit (GE Healthcare and Life Science). Relative density of immunoblots was analyzed by ImageJ software as described [23].

### Immunofluorescence

Sections were incubated with overnight at 4 °C in the following antibodies MPO (1:500, Abcam) and then incubated with appropriate fluorescence conjugated secondary antibodies (Jackson Immunoresearch, West Grove, PA, USA). A fluorescent Olympus-BX51 microscope was used to image immunofluorescence in MagnaFire SP 2.1B software (Olympus, Melville, NY, USA). At least six sections were counted in every animal group in a microscopic field of × 20 and expressed cells/field as previously described [24].

### Dihydroethidium (DHE) and hydroxyphenyl fluorescein (HPF) staining

Sections were incubated with DHE (2 μmol/L, Thermo Scientific) at 37 °C for 30 min in the dark. For HPF staining, sections were incubated at 37 °C (protected from light) for 1 h with HPF (Thermo Scientific). A fluorescence microscope (Olympus, Melville, NY, USA) was used to measure fluorescence. Each animal group was counted using at least six sections. To evaluate superoxide (O_2_^−^) and peroxynitrate (ONOO^−^), relative fluorescence intensity of DHE and positive HPF cells expressed cells/field in microscopic field of ×20 were measured by ImageJ (NIH).

### TUNEL staining

10-μm-thick sections were assessed according to the manufacturer’s protocol (Thermo Scientific). TUNEL staining was visualized by a fluorescence microscope (Olympus). ImageJ was used to evaluate average number of TUNEL-positive cells with at least six sections in each animal group.

### Nitric oxide assay

All procedures were performed following manufacturer’s protocol of nitric oxide assay kit (Abcam). The homogenizer (Tissue Miser Homogenizer; Fisher Scientific, Pittsburgh, PA, USA) was used to homogenize fresh tissues on ice. After collecting supernatant in a clean tube, perchloric acid (PCA, Fisher Scientific) was added in the tube for a final concentration of 1 M followed by incubation for 5 min. Then transfer the supernatant to a clean tube after centrifuge, adding an equal PCA volume of potassium hydroxide (KOH, Fisher Scientific) into the tube. Collecting supernatant after centrifuge for reaction wells preparation. A microplate colorimetric reader (OD540 nm, Bio-Rad, Hercules, CA, USA) was used to analyze absorbance. The NO volume was calculated dependent on a nitrite standard calibration curve.

### Statistical analysis

In this study, all animals were randomly assigned to different groups. All the experimental tests were blinded to the surgeons and researchers who did the experiments and analyze the research data. All tests for exploratory studies were performed two-sided. GraphPad was used to exclude outliers. Samples size were estimated using a type I error rate of 0.05 and a power of 0.8 on a 2-sided test by power analysis. For parametric data, analysis was using one-way ANOVA followed by the Tukey’s post hoc test. For non-parametric data, Kruskal-Wallis with Dunn’s post hoc tests was used to analysis. Longitudinal data were analyzed by two-way ANOVA with Tukey’s post hoc test. Data were expressed as mean ± SD. *P* values of <0.05 were considered statistically significant. GraphPad Prism 6 (La Jolla, CA, USA) and SigmaPlot 11.0 (SysStat, Germany) were used for graphing and analyzing all the data.

## Results

### BLVRA treatment attenuated short-term neurological deficits and splenic contraction up to 3 days after GMH

**Fig. 1:**
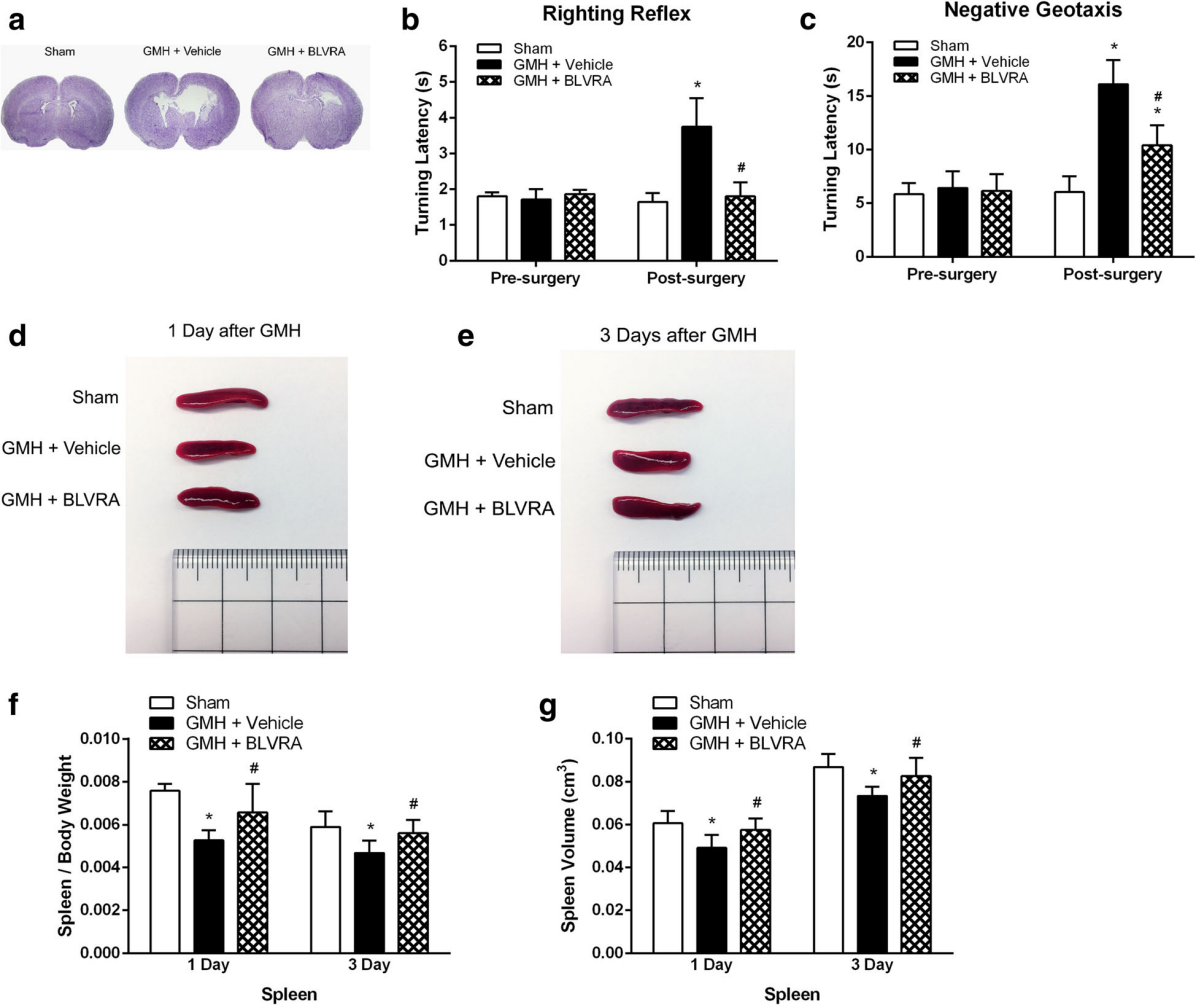
Effects of BLVRA on GMH-induced developmental delay and splenic atrophy. **a** Ventricular dilation at 3 days after GMH. Neurological assessments at 1 to 3 days after GMH using **b** righting reflex and **c** negative geotaxis. GMH induced splenic atrophy at **d** 1 and **e** 3 days after GMH. **f** Spleen to body weight ratio of sham with GMH rats and **g** splenic volume at 1 and 3 days after GMH. Values are expressed as mean ± SD. ^*^P < 0.05 compared with sham, ^#^*P* < 0.05 compared with GMH + vehicle. *N* = 6 each group

Brain morphology was evaluated by Nissl staining on day 3 after GMH. Ventricular volume was significantly increased in the vehicle group compared with sham animals, but BLVRA treatment decreased GMH-induced ventricular dilation (Fig. 1a). 3 days post-GMH, developmental delay was observed in the vehicle group compared with the sham group, as demonstrated in both righting reflex and negative geotaxis. BLVRA administration attenuated neurological deficits after GMH (Fig. 1b, c). Then we also observed that GMH induced significant splenic contraction on both day 1 and day 3 after GMH. BLVRA-treated animals had significantly less splenic contraction compared with the vehicle group (Fig. 1d, e). Our data on the ratio of spleen weight over body weight and spleen volumes also indicated that GMH induced significant splenic atrophy on days 1 and 3 post-ictus and that BLVRA effectively attenuated this atrophic response (Fig. 1f, g).

### The effect of GMH inducing splenocyte mobilization into the brain and splenic apoptosis were decreased by BLVRA treatment

**Fig. 2:**
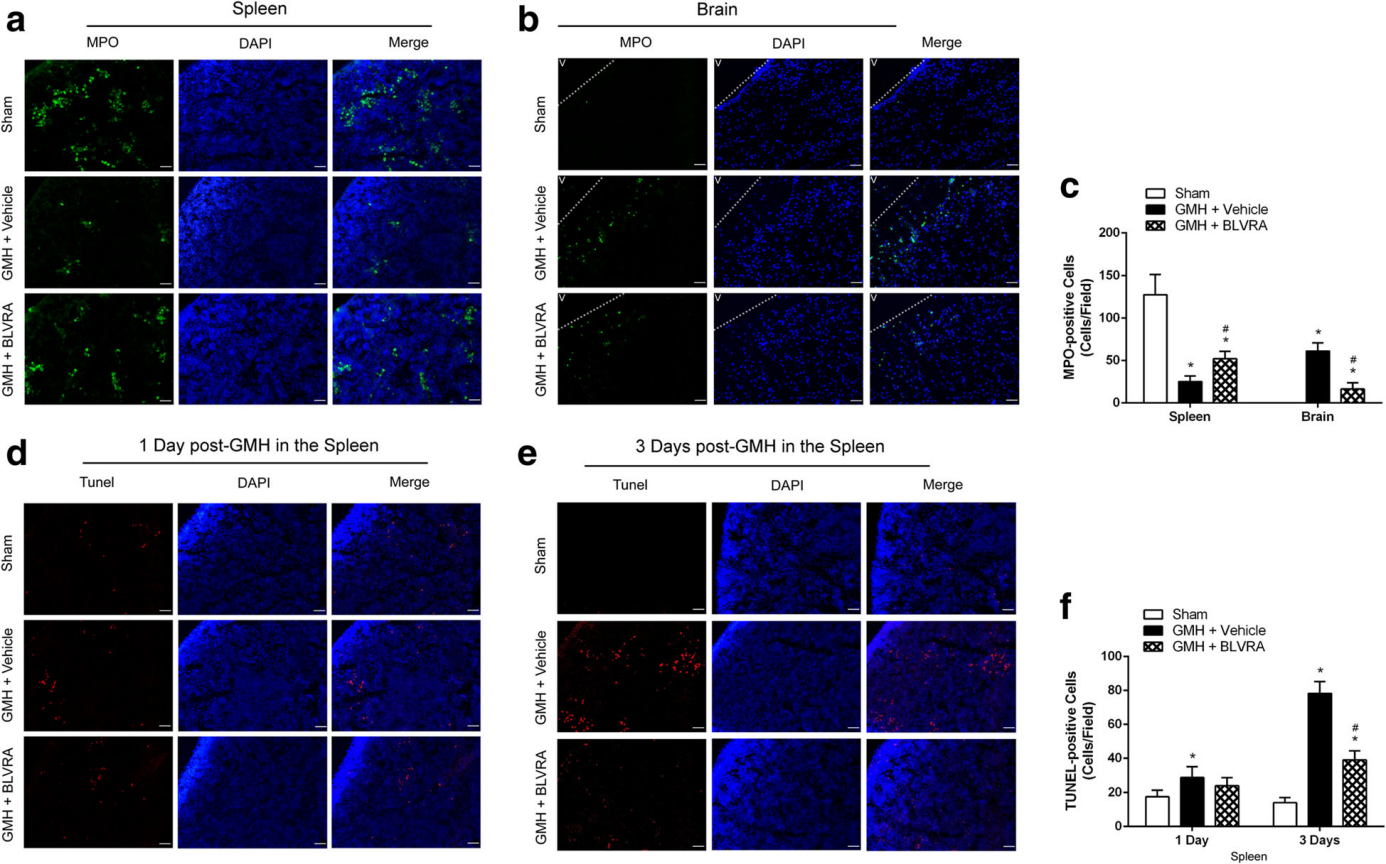
The role of BLVRA in GMH-induced neutrophil mobilization and splenic apoptosis. **a** Neutrophils from the spleen and **b** brain tissues in sham, vehicle, and BLVRA-treated animals at 1 day after GMH. **c** Quantification of neutrophils for MPO staining. Numbers of GMH-induced splenic cell death at 1 (**d**) and 3 (**e**) days after GMH. **f** Quantification of apoptotic splenic cells for TUNEL staining. Values are expressed as mean ± SD. ^*^P < 0.05 compared with sham, ^#^*P* < 0.05 compared with GMH + vehicle. *N* = 6 each group. N.D. = not detected

Neutrophils, monocytes, and natural killer cells play a critical role in the innate immunity [25–28]. Neutrophils exist in the spleen when the fetus is developing. It is defined as cleaning cells, offering the first response to immune challenges [25]. In order to determine whether spleen-derived peripheral immune cells accumulated in the brain, we evaluated neutrophils in the spleen and the brain 1 day after GMH. Immunofluorescence showed that GMH reduced the number of neutrophils in the spleen on day 1 (Fig. 2a). We further evaluated neutrophil infiltration into the brain 1 day after GMH. A larger number of neutrophils were observed in the peri-ventricular region of the brain (Fig. 2b). This induction was attenuated by BLVRA administration (Fig. 2a, b). Moreover, apoptosis was evaluated by TUNEL staining in the spleen on days 1 and 3 after GMH. A moderate increase in TUNEL-positive splenocytes was found on day 1 after GMH (Fig. 2d, f). Moreover, 3 days after GMH, the number of TUNEL-positive splenocytes strikingly increased (Fig. 2e, f). These results indicated that the reduction of splenocytes after GMH was probably due to their mobilization into the circulation, rather than splenic apoptosis on day 1 after GMH.

### BLVRA attenuated both splenic and brain inflammatory responses after GMH

**Fig. 3:**
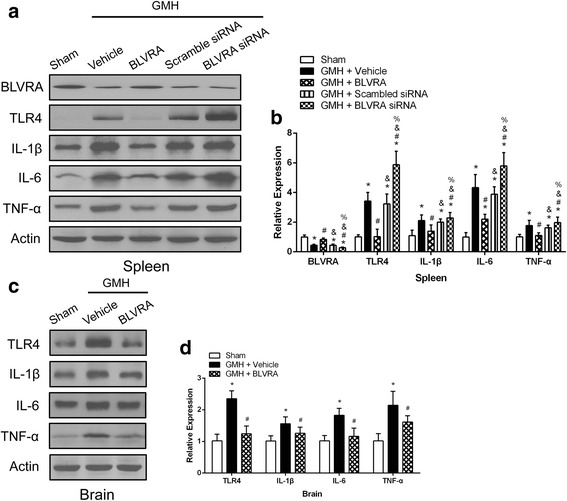
Knockdown of BLVRA aggravated GMH-induced inflammation. **a** Western blot analysis of BLVRA and inflammatory cytokine expression in BLVRA-treated and BLVRA-knockdown splenic tissues; representative blots are shown. **b** Quantitative analysis of BLVRA and inflammatory cytokine expression in BLVRA-treated and BLVRA-knockdown splenic tissues. **c** Western blot analysis of inflammatory cytokine expression in BLVRA-treated brain tissues; representative blots are shown. **d** Quantitative analysis of inflammatory cytokine expression in BLVRA-treated brain tissues. Values are expressed as mean ± SD. ^*^P < 0.05 compared with sham, ^#^*P* < 0.05 compared with GMH + vehicle. ^&^*P* < 0.05 compared with GMH + BLVRA. ^%^*P* < 0.05 compared with GMH + scrambled siRNA. *N* = 6 each group

Inflammation plays a major role in mediating secondary brain injury after GMH. Additionally, TLR4 is an important regulator of the innate immune system. Therefore, western blot was conducted to determine the TLR4-related immune response in the spleen and brain after GMH. We used BLVRA siRNA to knock down BLVRA in the spleen, leading to a significant reduction in its protein expression. With the knockdown of BLVRA, the splenic inflammatory profile demonstrated significant upregulation of TLR4 and proinflammatory cytokines (Fig. 3a, b). A similar trend was observed in the brain. TLR4 and inflammatory cytokines were significantly increased after GMH, but suppressed by BLVRA administration in the brain (Fig. 3c, d).

### Endothelia nitric oxide synthesize (eNOS) suppression adversely affect the beneficial effect of BLVRA in reducing splenic immune responses

It has been reported that biliverdin stimulation could induce phosphorylation of eNOS in macrophages in a BVR-dependent manner [21]. However, the relationship between BLVRA and phosphorylation of eNOS has barely been studied in the context of brain injuries.

**Fig. 4:**
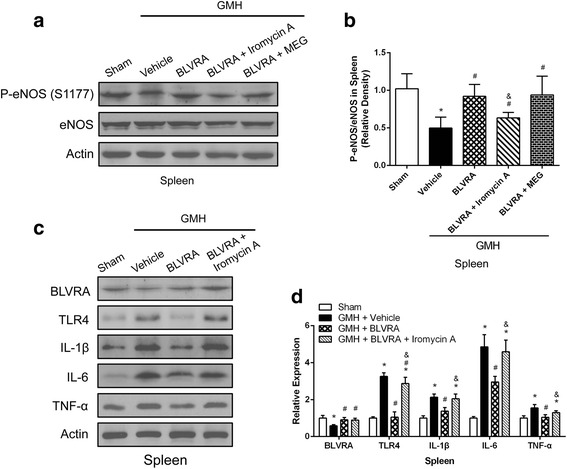
Inhibition of eNOS in BLVRA-treated rats abrogated the anti-inflammatory effects induced by BLVRA. **a** Western blot assay was used to analyze the expression of phosphorylated eNOS and eNOS in splenic tissues in BLVRA, BLVRA + eNOS inhibitor and BLVRA + iNOS inhibitor animals. **b** Quantitative analysis of phosphorylated eNOS and eNOS in spleen tissues in BLVRA, BLVRA + eNOS inhibitor and BLVRA + iNOS inhibitor animals. **c** Western blot analysis of inflammatory cytokine expression in BLVRA and BLVRA + eNOS inhibitor groups. **d** Quantitative analysis of inflammatory cytokine expression in BLVRA and BLVRA + eNOS inhibitor groups. Values are expressed as mean ± SD. ^*^P < 0.05 compared with sham, ^#^*P* < 0.05 compared with GMH + vehicle. ^&^*P* < 0.05 compared with GMH + BLVRA. *N* = 6 each group

We hypothesized that phosphorylation of eNOS might be a downstream effector of BLVRA. Thus, we first measured phosphorylated eNOS after GMH in the spleen. The expression level of phosphorylated eNOS was significantly increased in response to BLVRA stimulation after GMH, indicating a potential connection between BLVRA and eNOS phosphorylation (Fig. 4a, b). To further assess the effect of phosphorylated eNOS on inflammation in the presence of BLVRA, we measured the protein levels of TLR4 and inflammatory cytokines in eNOS-suppressed spleens. GMH elicited significant splenic inflammation, which was attenuated by BLVRA. However, the beneficial effects of BLVRA in reducing splenic immune responses after GMH were abolished by eNOS inhibitor Iromycin A (Fig. 4c, d).

### BLVRA translocated to the nucleus in an eNOS/NO dependent manner

**Fig. 5:**
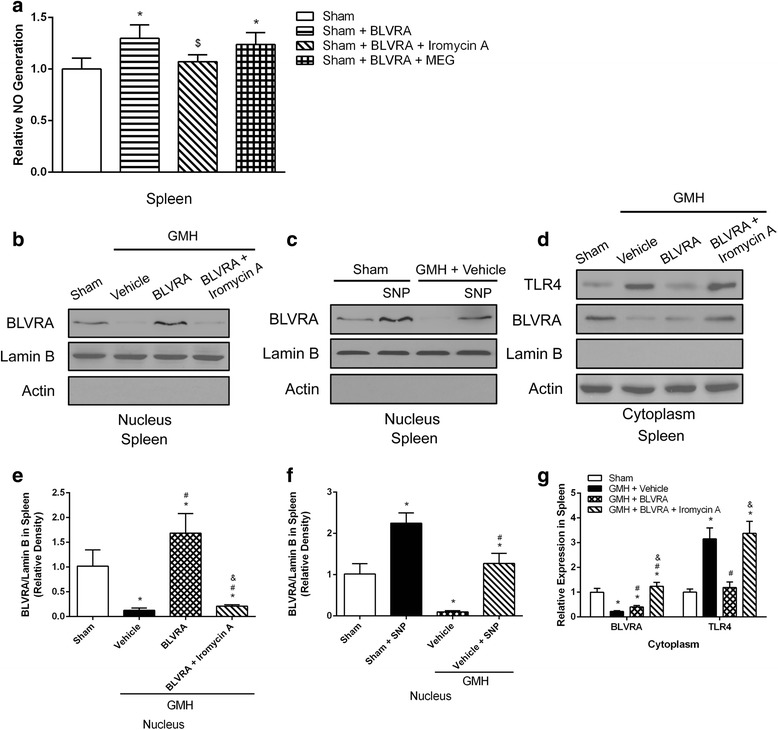
BLVRA nuclear translocation was dependent on eNOS-derived NO. **a** NO production was measured in splenic tissues treated with BLVRA and co-administered with eNOS inhibitor and iNOS inhibitor, respectively. Western blot analysis of BLVRA expression in the nucleus of spleen tissues treated with BLVRA + eNOS inhibitor (**b**), BLVRA + NO donor (SNP) (**c**). **d** Western blot analysis of BLVRA and TLR4 expression in the cytoplasm treated with BLVRA and BLVRA + eNOS inhibitor. **e**–**g** Representative quantitative analysis were shown respectively. Values are expressed as mean ± SD. ^*^P < 0.05 compared with sham, ^#^*P* < 0.05 compared with GMH + vehicle. ^&^*P* < 0.05 compared with GMH + BLVRA. *N* = 6 each group

As BVR directly binds to AP-1 sites of TLR4 promoter, it inhibits TRL4 expression [21]. Inhibition of eNOS abolished BLVRA-mediated suppression of TLR4. We next investigated whether eNOS was involved in the inhibitory effect of BLVRA on TLR4 expression. BLVRA induced significant increase in NO generation, in accordance with eNOS phosphorylation but independent of iNOS, since no effect of NO generation was found in response to BLVRA administration after co-administration with iNOS inhibitor MEG (Fig. 5a). Furthermore, BLVRA administration resulted in accumulation of BLVRA in the nucleus, which was eliminated with eNOS inhibition (Fig. 5b, e). A similar trend was found with the administration of NO donor in the absence of BLVRA stimulation, which also led to significant accumulation of BLVRA in the nucleus (Fig. 5c, f). In addition, cytoplasmic BLVRA level was suppressed after GMH but significantly higher with the co-administration of BLVRA and Iromycin A. Cytoplasmic TLR4 was also induced by GMH and attenuated by BLVRA administration. Interestingly, the co-administration of Iromycin restored the TLR4 expression to a similar level to the vehicle group. Thus, NO generated from eNOS seemed to be responsible for translocating BLVRA into the nucleus and inhibiting TLR4 expression.

### BLVRA reduced oxidative and nitrosative stress induced by GMH

**Fig. 6:**
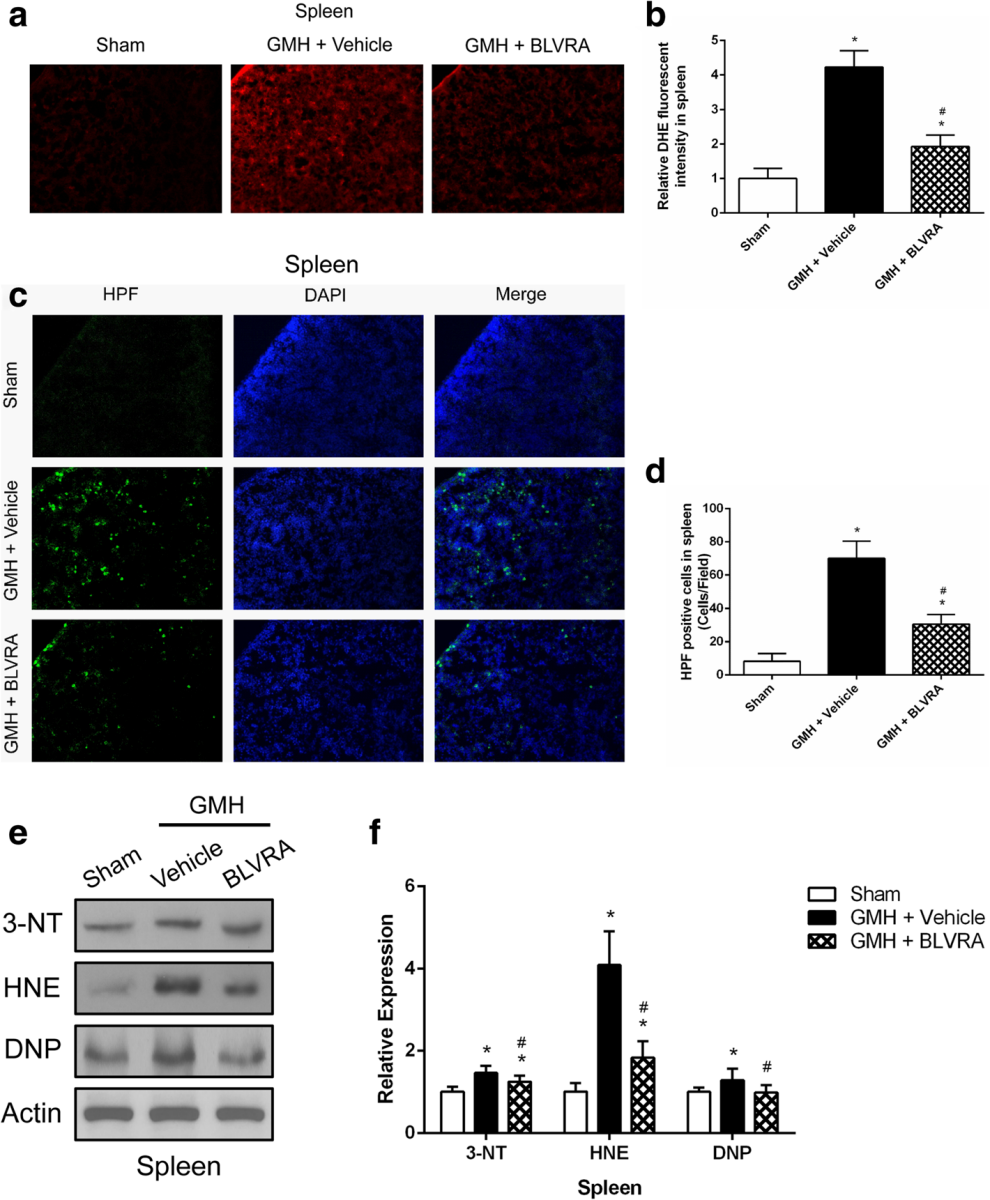
BLVRA attenuated oxidative stress and nitrosative stress. **a**, **b** Image and quantification of superoxide in GMH animals treated with BLVRA for DHE staining. **c**, **d** Image and quantification of peroxynitrate in GMH animals treated with BLVRA for HPF staining. **e** Western blot assay was used to characterize 3-NT, HNE, and DNP expression in BLVRA-treated spleen tissues. **f** Quantification analysis of 3-NT, HNE, and DNP expression in BLVRA-treated spleen tissues. Values are expressed as mean ± SD. ^*^P < 0.05 compared with sham, ^#^*P* < 0.05 compared with GMH + vehicle. ^&^*P* < 0.05 compared with GMH + BLVRA. *N* = 6 each group

Since oxidative stress is strongly correlated with inflammation, and BLVRA is a physiologic antioxidant, GMH-induced oxidative stress and nitrosative stress were assessed by staining and western blot. BLVRA administration resulted in a significant decrease in superoxide (O_2_^−^) and peroxynitrite (ONOO^−^) in the spleen (Fig. 6a–d). Oxidative and nitrosative stress biomarkers (3-NT, HNE, and DNP) in the splenic tissues were also significantly reduced after BLVRA administration (Fig. 6e, f).

## Discussion

GMH is a common brain injury of premature infants. The resultant splenic response is thought to play an important role in worsening post-hemorrhagic brain injury [29, 30]. In clinical settings, immunodepression was found within days after stroke, characterized by lymphopenia, increased anti-inflammatory cytokines, and splenic contraction [31]. These observations indicated a systemic inflammation generated immediately after stroke. Infections, such as pneumonia and urinary tract infection, have high incidence in stroke patients, which may even further increase their mortality. Therefore, it is crucial to understand immune responses after GMH, since neonates have limited adaptive immunity. Spleen is a secondary peripheral immune organ that reserves large quantities of immune cells to produce pro-inflammatory responses after brain injuries [5]. Therefore, we assessed the stored peripheral immune cells released into the circulation as the splenic immune responses to GMH. Indeed, in this study, we explored how BLVRA ameliorated GMH-induced brain injuries and the consequent neurological deficits. We further evaluated the underlying mechanisms by which BLVRA attenuated inflammatory responses in the spleen. It might be via direct inhibition of TLR4, a key mediator of innate immunity and attenuation of the infiltration of peripheral immune cells.

We used a collagenase GMH neonatal model to mimic the motor deficits and ventricular dilation as observed in human premature infants [22]. The underlying mechanisms leading to these consequences remain to be elucidated, which is the main purpose of the present study. Consistent with previous studies, we found significantly developmental delay in GMH rats, with spontaneous resolution of neurological deficits 3 days after GMH [22, 32]. In our previous study, GMH caused a dramatic decrease in the endogenous BLVRA expression level in the brain from 3 h after GMH. The consumption of BLVRA may contribute to activating microglia and resolving hematoma by translocating into the nucleus and inhibiting the TLR-4 promoter. GMH-induced reduction of BLVRA was increased after 1 day, but still lower than the sham level. The reason for the decrease may be that the profound inflammatory response from GMH consumes BLVRA accumulated in the brain. The expression levels of inflammatory cytokines were striking increased after GMH, which cause the continuous consumption of BLVRA more than its accumulation [33].

BLVRA treatment attenuated short-term neurological deficits GMH animals. Neonates with GMH demonstrated an initial period of splenic atrophy starting on day 1 post-ictus. Spleen weight/body weight ratio and spleen volume were both reduced in BLVRA-treated pups. These differences in GMH neonates on day 3 were considerably smaller than day 1 compared with sham animals. A potential mechanism by which the spleen might promotes neurodegeneration after stroke is through the activation of sympathetic nervous system, contributing to splenic atrophy and release of peripheral immune cells [34]. These proinflammatory immune cells infiltrate into the brain and worsen neuroinflammation, as well as neurodegeneration [7].

Neutrophils, monocytes, and natural killer cells make up the innate immune system. Neutrophils primarily migrate to the inflammatory site, and infiltrate via the cerebral microvasculature, causing blood flow breakdown and brain damage [35–37]. We demonstrated a marked decrease in neutrophils in the spleen in response to GMH, which was reversed by BLVRA treatment. BLVRA also attenuated neutrophil infiltration into the brain 1 day after GMH. The reduction of splenic immune cells is unlikely due to cellular apoptosis or differentiation to macrophages/dendritic cells. Instead, it might result from cellular mobilization into the circulation and to inflamed tissues [38]. However, a dramatic increase in apoptotic response occurred on day 3, rather than day 1 post-GMH. According to a previous report, by 96 h after stroke, the percentage of B cells was reduced by about half to ~ 30% of the remaining splenic cells. According to the research [39], we presume that the reduction of B cells by 1 day constituted the majority of splenocytes loss, as well as a striking decrease of B cells by 3 days. These changes were accompanied by an increase in splenocytes that were committed to the apoptotic event showed by TUNEL staining. This is consistent with studies showing significant splenic cell death after focal cerebral ischemia, which is likely to occur in hyporesponse to T cell mitogens stimulation at 96 h after brain injury [39].

TLR4 is a typified TLR family member which is strongly related to the innate immune system by triggering downstream pathway to activate transcription of pro-inflammatory genes [40]. A profound increase in TLR4 and inflammatory cytokines, including IL-1β, IL-6, and TNF-α, was demonstrated in the GMH-injured brain. These effects were attenuated by BLVRA treatment. Since biliverdin reductase function as inflammatory regulator by directly binding to the AP-1 sites of pro-inflammatory effectors, such as TLR4 [21]. Thus, our results showed that BLVRA knockdown exacerbated splenic inflammation, indicating that BLVRA was important in reducing GMH-induced splenic inflammatory responses.

It has been reported that treatment in macrophages with biliverdin led to a rapid increase in eNOS phosphorylation through a biliverdin reductase-dependent mechanism [21]. Then, we tested whether BLVRA was present and regulated eNOS phosphorylation in the spleen. The marked increase in phosphorylation of eNOS was observed with BLVRA treatment. The BLVRA-suppressed splenic inflammation was dependent on eNOS, since a selective eNOS inhibitor, Iromycin A, abolished the beneficial effects of BLVRA. The marked increase in phosphorylation of eNOS was consistent with increased NO generation. In addition, no significant changes in NO production in response to BLVRA stimulation was seen with the selective iNOS inhibitor, MEG, indicating that this mechanism of BLVRA on NO generation was eNOS-dependent. NO signaling was involved in regulating the immune system through S-nitrosylation of posttranslational modification [21]. Furthermore, S-nitrosylation, a form of cysteine modification interacting with enzymatic function, is prominent in regulating immune responses, such as TLR4 signaling [41, 42]. Since LPS induced BLVRA S-nitrosylation, NO might modify BLVRA on a cysteine, leading to its nuclear translocation and the consequent immune regulation. The role of BLVRA in regulating NO generation and the mechanism by which GMH induced splenic inflammation have not been described. We hypothesized that BLVRA modulated eNOS-derived NO and NO, in return, translocated BLVRA into the nuclear, resulting in suppressed inflammatory responses. This hypothesis is strengthened by accumulation of BLVRA in the nucleus of splenocytes with BLVRA administration, which was correlated with increased phosphorylation of eNOS and NO generation. This effect is performed to be NO dependent, because similar BLVRA accumulation in the nucleus was observed after treatment with NO donor, SNP, without BLVRA stimulation. eNOS inhibitor led to decreased BLVRA translocation to the nucleus. Suppression of TLR4 protein was consistent with BLVRA translocation. Thus, splenic inflammatory responses were mediated through the eNOS/NO signaling.

GMH induced inflammatory responses, characterized by concomitant free radicals causing oxidative stress responsible for neurodegeneration in neonates. A redox cycle of bilirubin provided cytoprotection dependent on BLVRA has been investigated in a recent study [43]. When BLVRA was stimulated, the degree of oxidative stress and nitrosative stress was greatly reduced in the spleen. The known physiological event that administration of BLVRA attenuated oxidative stress might depend on the strong antioxidant potential of bilirubin.

Although we extensively examined the splenic response in this study, intranasal administration of BLVRA did not provide evidence of its direct effects on the spleen. Systemic administration of BLVRA would have demonstrated this direct connection. Vice versa, although we validated this signaling pathway in the brain both in this study and in a previous report [33], it is still difficult to dissociate the local beneficial effects of BLVRA from its suppressive effects on peripheral immune response. Thus, splenectomy could be a potential strategy to dissociate these two effects.

## Conclusions

In summary, we have identified a BLVRA-dependent mechanism regulating inflammatory responses and splenocyte death after GMH. We also provided evidence to support BLVRA translocating to the nucleus to suppress TLR4 expression. BLVRA-induced increase of phosphorylation of eNOS in the spleen is an important pathway for modulating inflammatory responses in the spleen after GMH.

## Abbreviations

BLVRA: Biliverdin reductase-A
BVR: Biliverdin reductase
GMH: Germinal matrix hemorrhage
HO: Heme oxygenase
NOS: Nitric oxide synthase
TLR: Toll-like receptor
